# Multiscale Modification of *Populus cathayana* by Alkali Lignin Combined with Heat Treatment

**DOI:** 10.3390/polym10111240

**Published:** 2018-11-09

**Authors:** Haizhen Zhou, Jingyu Li, Erni Ma

**Affiliations:** Beijing Key Laboratory of Wood Science and Engineering, MOE Key Laboratory of Wooden Material Science and Application, Beijing Forestry University, Qinghua Eastroad 35#, Haidian, Beijing 100083, China; zhzbjfu@163.com (H.Z.); ljyemma@163.com (J.L.)

**Keywords:** wood modification, alkali lignin, water resistance, dimensional stability, heat treatment

## Abstract

Chemical modification of wood with green modifiers is highly desirable for sustainable development. With the aim of enhancing the water resistance and dimensional stability of fast growing wood, modifications were conducted using renewable and toxicity-free industrial lignin combined with heat treatment. Poplar (*Populus cathayana*) samples first underwent impregnation with alkali lignin solution and were then subjected to heat treatment at 140–180 °C for two hours. The results indicated that the modified wood showed excellent leaching resistance. The alkali lignin treatment improved surface hydrophobicity and compression strength, and decreased moisture and water uptake, thereby reducing the dimensional instability of modified wood. These changes became more pronounced as the heat-treating temperature increased. Scanning electron microscopy, confocal laser scanning microscopy, and Fourier transform infrared spectroscopy evidenced that a multiscale improvement of the alkali lignin occurred in the cell lumen and cell wall of wood fibers and vessels, with small alkali lignin molecules reacting with the wood matrix. This study paves the way for developing an effective modification approach for fast growing wood, as well as promoting the reuse of industrial lignin for high-value applications, and improves the sustainable development of the forestry industry.

## 1. Introduction

As the second richest polymer from biomass after cellulose, lignin is usually accepted as biomass waste in the pulping industry. The lack of use of lignin has led to a massive waste of resources and pulping emissions have become an issue of environmental concern [1]. Only about 100 kt (less than 2%) can be sold commercially as kraft or alkali lignins [2].

Lignin is regarded as a natural raw material that can modify or replace other materials because of its unique chemical structure and abundance in nature. Peng et al. found that polypropylene composites reinforced with alkali lignin showed higher surface hydrophobicity after a weathering treatment [3]. Directly using industrial lignin and modified lignin as fillers was found to lead to a significant reduction in water absorption in thermoplastic starch [4]. Composites mixed by wood flour, lignin (extracted from kraft black liquor) and polypropylene showed decreased hydroscopicity and corresponding swell rate with increasing lignin content [5]. Therefore, lignin is thought to make the composites repellent to water and deformation. However, few studies in the literature examined lignin as an alternative to enhance the hydrophobicity and dimensional stability of solid wood whose hygroscopic nature of the cell wall polymers is a dominating obstacle to the application of usual hydrophobic modifiers. The poor reaction capacity of lignin at room temperature also limits the modification effect of wood.

The problem may be solved by heat treatment. Studies have shown that lignin chemical reactivity increased during heat treatment, since a number of reactions including radical oxidation, molecular transposition, dehydrogenation, and polycondensation occurred, resulting in cross-links between lignin molecules [6,7,8]. Due to oxidation and condensations, a further self-crosslinking occurs between lignin and the cell wall components of wood when heating [9]. On the other hand, the porosity of wood cell wall increases after heat treatment due to the degradation of hemicellulose [10], which provides the possibility of improving permeability for lignin. Heat treatment has been proven to boost water repellency and dimensional stability of wood caused by decreasing free hydroxyl [11,12]. Therefore, heat treatment could be a good consideration to combine with lignin modification. And according to the previous research [13], heat treatment with lower temperature or short heating time did not adversely affect the mechanical properties of wood.

The objective of this study was to take industrial lignin as a modifier to enhance the water resistance and dimensional stability of fast growing wood. Heat treatment was introduced to facilitate a redistribution of the impregnated lignin and cross-links between it and wood matrix. The effects of this combined treatment, including water resistance and dimensional stability, were investigated. Morphological and chemical characteristics were analyzed by scanning electron microscopy (SEM), confocal laser scanning microscopy (CLSM), and Fourier transform infrared (FTIR) spectroscopy, with high expectation of multiscale modifying both the cell cavity and cell wall levels. Results from this study expand the application field and resolve the bottleneck for the use of fast growing wood, and enable the high-valued reuse of industrial lignin, therefore contributing to more environmentally friendly and sustainable development of the forestry industry.

## 2. Materials and Methods

### 2.1. Materials

The test species was Poplar (*Populus cathayana*), which belongs to the fast growing species, and is quite common in North China. The 20 × 20 × 20 mm^3^ (L × T × R) samples were oven-dried at 103 °C until they reached a constant weight *m*_0_ (g) (labelled as Control). Alkali lignin extracted from pulping black liquor (average size of 180 nm detected by a laser particle size analyzer, MASTERSIZE 2000, Malvern Panalytical Ltd., Malvern, England and number-average molecular weight of 1336 g/mol obtained by gel permeation chromatography, Agilent 1200, Agilent Technologies Inc., Santa Clara, CA, USA), was purchased from Shan Feng Chemical Co., Ltd., Changzhou, China.

### 2.2. Sample Treatment Methods

The alkali lignin particles were dispersed in 1,4-dioxane to the maximum concentration followed by filtration for later use. The samples were impregnated with alkali lignin solution using a vacuum-pressure process. In this study, the samples were subjected to a vacuum at −0.1 MPa for 30 min, then they were immersed in alkali lignin solution at 0.5 MPa for 1 h. After impregnation, the excess liquid in the wood surface was wiped with tissue paper and oven-dried at 103 °C to constant weight *m*_1_ (g) (labelled as AL). Finally, the AL samples were subjected to three temperatures of 140, 160, and 180 °C for 2 h (labelled as AL 140 °C, AL 160 °C, and AL 180 °C, respectively), and the weight *m*_2_ (g) after heat treatment was recorded. The weight percent gain (WPG) for the impregnation treatment and mass loss (ML) for the heat treatment were evaluated according to the following equations:

WPG(%) = 100(*m*_1_ − *m*_0_)/*m*_0_ (1)

ML(%) = 100(*m*_1_ − *m*_2_)/*m*_1_ (2)

### 2.3. SEM Analysis

To characterize structural details and the alkali lignin location of the modified wood, transverse and tangential sections of the samples were placed in conductive glue and sputter-coated with gold. Then, the microstructure of the samples was observed by SEM (Hitachi S-3400, Hitachi Ltd., Tokyo, Japan) with a voltage of 15 KV.

### 2.4. CLSM Analysis

To further qualitatively and quantitively investigate the spatial distribution of alkali lignin within the samples, transverse sectional samples 10–20 μm thick from the microtome were subsequently inspected with a Leica TCS SP5 confocal microscope (Leica Microsystems Inc., Wetzlar, Germany) with a krypton/argon laser emitting at wavelengths of 488 nm. The same gain setting (115) was used for the samples so the emission intensities could be directly compared in the images.

### 2.5. FTIR Spectroscopy Analysis

To explore the changes in the functional groups in the wood after treatment, flour of the samples (100 mesh) was pressed into KBr pellets and tested by FTIR (Bruker VERTEX 70V, Bruker Ltd., Karlsruhe, Germany) with a resolution of 4 cm^–1^ and 32 scans per sample in the 4000–400 cm^–1^ interval.

### 2.6. Leachability Test

The treated samples were tested for leaching resistance according to the E11-06 American Wood Protection Association (AWPA) standard. For each group, three replicates were leached in 300 mL of deionized water at 22–28 °C. The samples were completely submerged and stirred with a magnetic stir bar in flask bottles. The water was replaced after 6, 24, and 48 h, and thereafter at 48-h intervals. The leachability test lasted for 14 days, and the weights of the samples (dried at 103 °C) before and after the leaching treatment were recorded.

### 2.7. Contact Angle

All the samples were conditioned at 25 °C and 72% relative humidity (RH) to a constant mass before measurement. The time-dependent contact angles were measured according to sessile drop method by Dataphysics OCA20 contact angle analyzer (DataPhysics Instruments GmbH, Filderstadt, Germany). Data were gathered stochastically from three sites on the transverse section of every sample with each group containing three replicates. The dispense process was completed by an automatic microsyringe with approximately 3 μL of distilled water.

### 2.8. Water Resistance and Dimensional Stability

Water resistance and dimensional stability were tested according to Chinese standard GB/T 1934.1-2009 and GB/T 1934.2-2009, respectively. Oven-dried samples were exposed separately to 3 different RH of 45%, 55%, and 72% at 25 °C for adsorption. The 3 RH conditions were created by applying saturated salt solutions of K_2_CO_3_, NaBr, and NaCl, respectively [14]. The remaining oven-dried samples were submerged in deionized water at approximately 25 °C for water absorption. The water used in the experiment was replaced daily to ensure the container stayed clean. Weights and dimensions (tangential, radial, and longitudinal direction) were examined at particular intervals during the adsorption and absorption processes. The moisture content (MC), water absorption rate (WAR), tangential swelling rate (α_w_ and α_max_), and volumetric swelling rate (α_Vw_ and α_Vmax_) of the samples were calculated from three replicates as follows:

MC(%) = 100(*M_n_* − *M*_0_)/*M*_0_ (3)

WAR(%) = 100(*W_n_* − *M*_0_)/*M*_0_ (4)

α_w_(%) = 100(*L_w_* − *L*_0_)/*L*_0_ (5)

α_max_(%) = 100(*L_max_* − *L*_0_)/*L*_0_ (6)

α_Vw_(%) = 100(*V_w_* − *V*_0_)/*V*_0_ (7)

α_Vmax_(%) = 100(*V_max_* − *V*_0_)/*V*_0_ (8)

where the subscript *w* represents the adsorption process; *max* refers to the absorption process; *M*_0_, *M_n_*, and *W_n_* are weights of over-dried samples and samples after *n* hours of adsorption and *n* hours of water absorption (g), respectively; *L*_0_, *L_w_*, and *L_max_* are the tangential dimensions of over-dried samples and samples after *n* hours of adsorption and *n* hours of water immersion (mm), respectively; and *V*_0_, *V_w_*, and *V_max_* are volumes of over-dried samples and samples after *n* hours of adsorption and *n* hours of water immersion (mm^3^).

The adsorption behavior of the modified samples was analyzed using the Hailwood–Horrobin (H–H) sorption model [15]. According to this theory, the MC corresponding to the total hydration of the available sorption sites (–OH) in wood was 18/W.

### 2.9. Compression Test

The samples were conditioned over a NaCl saturated solution (75% RH) at 25 °C before mechanical tests. The axial compressive strength was determined with a universal mechanical machine (Universal Mechanical Testing Machine, Beijing, China) at a test speed of 1 mm·min^−1^ according to GB/T 1935-2009. Each group included 6 replicates in the tests. The axial compressive strength (σ) was calculated as follows:

σ = *P_max_/bt* (9)

where *P_max_* is failure load (N), and *b* and *t* are the radial and tangential length of the samples (mm), respectively.

## 3. Results and Discussion

### 3.1. General Description of Treated Wood

As listed in Table 1, the WPG of the samples after impregnation ranged from approximately 11% to 13%. The mass loss increased with heat treatment temperature, since an increase in temperature promoted the degradation of hemicellulose. After the leachability test, all treated samples showed considerable leaching resistance, especially the AL 180 °C, for which the leaching rate was only 1.4%. This is ascribed to the hydrophobicity of alkali lignin and the formation of chemical bonds between alkali lignin and the wood matrix during heat treatment, which caused the fixation of alkali lignin in wood.

### 3.2. SEM Analysis of Treated Wood

**Figure 1:**
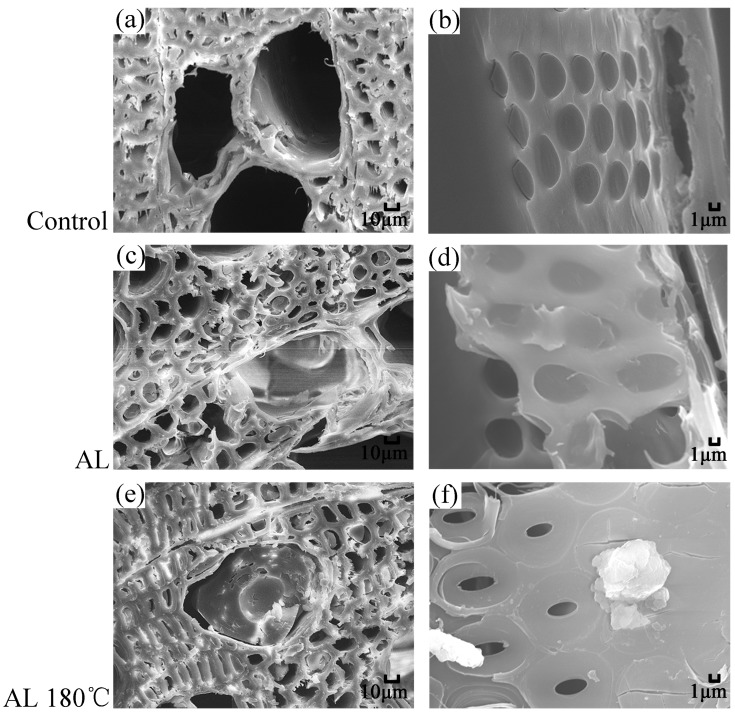
Scanning electron microscopy (SEM) images of (**a**,**c**,**e**) transverse-sections at magnification 500 and (**b**,**d**,**f**) tangential sections at magnification 3000 of the samples.

Figure 1 shows the SEM images of transverse and tangential sections of the samples. Compared with the Control, a portion of the cell cavity of wood fibers and vessels were totally or partially filled with alkali lignin after alkali lignin impregnation for the AL (Figure 1c). The fillers hinder water passage and may lead to further improvement in mechanical properties. The pits were covered by alkali lignin film (Figure 1d), which blocks the water paths in cell cavities and obstructs the possible hydrogen bonding contact between water molecules and hydroxyl groups in cell walls. After the heat treatment at 180 °C, granular sediments were found near the pits (Figure 1f), which may be associated with the self-condensation of alkali lignin caused by high temperature treatment. The cell wall of AL 180 °C showed no visible difference compared to the Control and the AL, implying that low heat treatment temperature would have little effect on the mechanical properties of wood.

### 3.3. CLSM Analysis of Treated Wood

**Figure 2:**
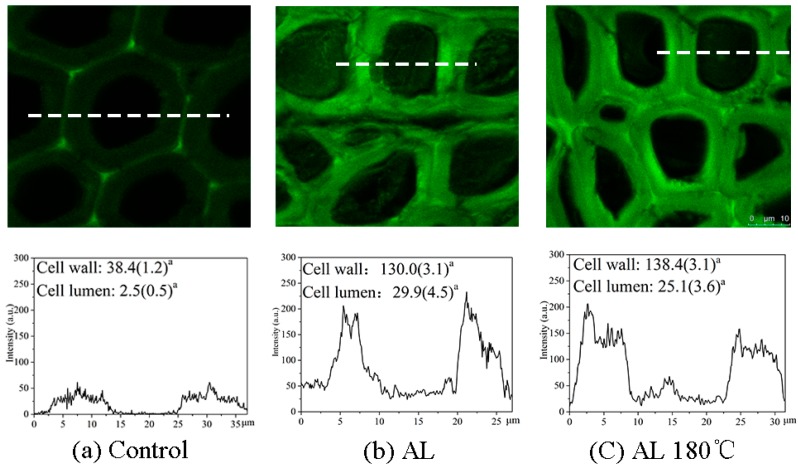
Confocal laser scanning microscopy (CLSM) images showing the lignin distribution over transverse sections of the (**a**) Control, (**b**) AL, and (**c**) AL 180 °C and corresponding varieties of fluorescence signal intensity across the wood cell along the white dotted lines. ^a^ Average values (standard deviation) from five replicates are shown.

Brighter areas represent higher lignin concentration. As shown in Figure 2, green light fluorescence was found in the cell wall of the Control with brighter fluorescence occurring in the compound middle lamella (CML), suggesting a higher lignin concentration in the CML [16]. The average fluorescence intensity was estimated by randomly calculating various area of the cell wall or cell cavity from five replicates, which also shown in Figure 2. The average intensity of the cell wall of the Control was 38.4, whereas that of the cell cavity was only 2.5. For AL and AL 180 °C, both the brightness of the image and fluorescence signal intensity increased significantly compared with the Control. The intensity values were higher than 130 for the cell wall and 25 for the cell cavity, which indicates that alkali lignin was not only deposited in the cell cavity, but also entered the cell wall after immersion. Further compared to the AL, we found from the signal intensity curve that the fluorescence intensity distribution was more homogeneous for AL 180 °C, suggesting a uniform dispersion of alkali lignin after heat treatment, which may have resulted from the increasing fusibility of alkali lignin caused by high temperature treatment [17].

### 3.4. FTIR Spectroscopy Analysis

**Figure 3:**
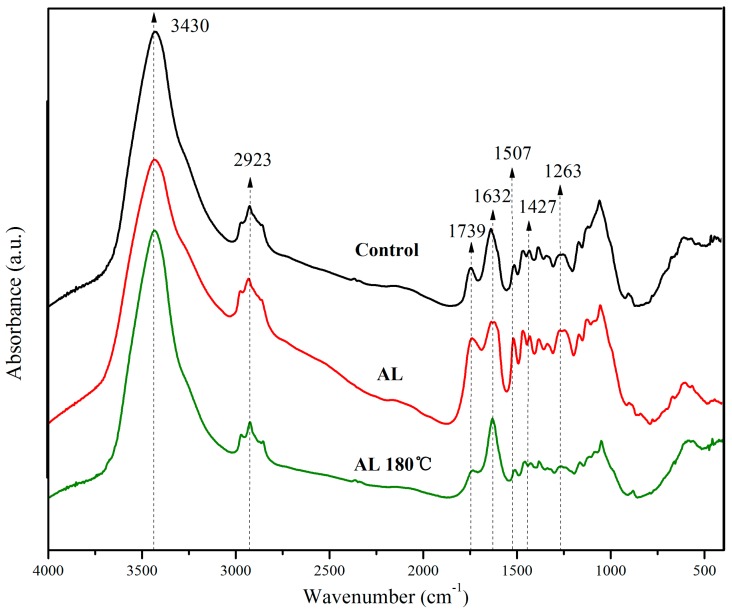
Fourier transform infrared (FTIR) spectra for the Control, AL, and AL 180 °C samples.

Taking the Control, AL, and AL 180 °C as examples, Figure 3 displays the FTIR spectra for the alkali lignin-heat treated samples, which were all normalized based on the highest peak assigned to O–H stretching around 3430 cm^–1^. Generally, analysis of changes in wood chemical components by O–H stretching absorption bands and C–H absorption bands (around 2923 cm^–1^) is limited, which is due to the fact that the three main chemical components (cellulose, hemicellulose, and lignin) of wood all contain hydroxyl and methylene moieties [18].

**Figure 4:**
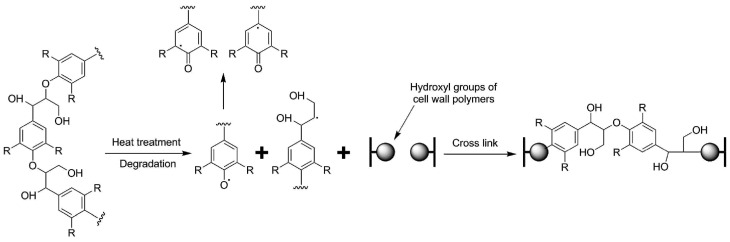
Schematic illustration and chemical reactions showing the bonding mechanism between lignin and the wood matrix.

From Figure 3, it could be found that the peak intensity in AL at 1632 and 1427 cm^–1^ increased significantly compared with the Control. The peaks were assigned to aromatic conjugated C=O and benzene ring vibration respectively, which originated mainly from lignin [19]. Another characteristic peak of lignin at 1507 cm^–1^ (C=C stretching vibration) [20] also increased after alkali lignin impregnation treatment, which is due to the infusion of alkali lignin in wood. Therefore, the relative amount of lignin for AL increased. The absorbance at 1263 cm^–1^ is the characteristic peak of Ar–O stretching or α- or β-aryl ether in lignin [21,22]. The rising intensity of this peak indicates new ethers formed between the alkali lignin or between alkali lignin and polysaccharide in AL (Figure 4), which also explains the low leaching rate of the treated samples because of the fixation of alkali lignin in wood (Table 1). The subdued signal at 1739 cm^–1^ assigned to C=O stretching, attributed primarily to hemicellulose [23], showed a decrease in the relative amount of hemicellulose for the AL 180 °C due to hemicellulose degradation caused by high temperature treatment.

Because the 1263, 1427, 1507, 1632 cm^–1^ wave numbers were attributed to the functional groups of lignin, to quantitatively estimate the change in lignin content or lignin functional groups after treatment, the lignin index (LI) was calculated according to Equation (10) based on the intensity of the peak at 1049 cm^–1^, which corresponds to the oxygen-containing functional groups of cellulose in wood [24], usually serving as a reference band.

LI = 100(*I*_1263,1427,1507,1632_/*I*_1049_) (10)

where *I* is band intensity.

As shown in Table 2, the LI_1263_, LI_1427_, and LI_1507_ of AL and AL 180 °C increased compared to the Control, again confirming the increase in the lignin content in treated wood. However, the values of LI_1263_, LI_1427_, and LI_1507_ of AL 180 °C were lower than those of AL, with an opposite trend for LI_1632_, which belongs to the aromatic conjugated C=O in lignin. This may be due to the degradation of alkali lignin after heat treatment (Figure 4), followed by the formation of aromatic organics [25,26].

### 3.5. Contact Angle

**Figure 5:**
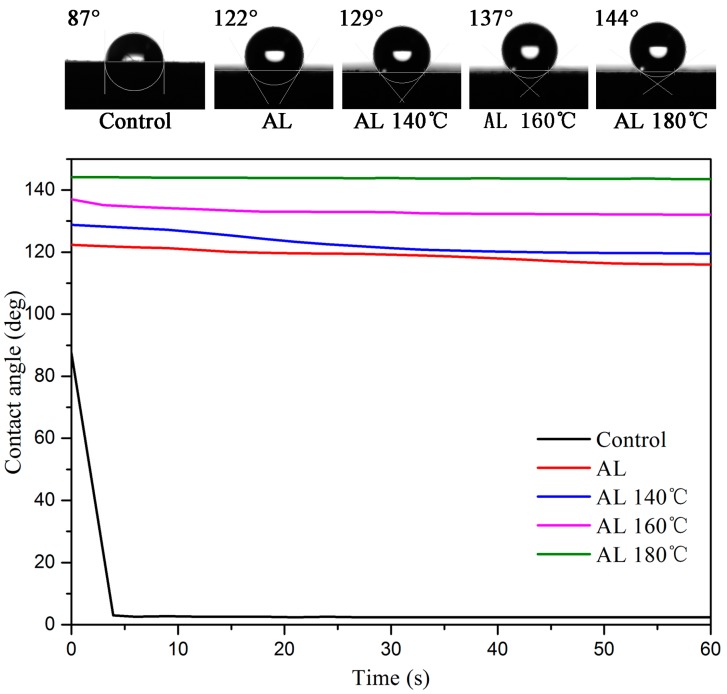
Initial contact angle and contact angle as a function of time for the samples.

The initial contact angle and change in contact angle with time for the samples is demonstrated in Figure 5. The contact angle of the Control was less than 90°, corresponding to a hydrophilic surface, and all the treated samples had contact angles greater than 90°, which indicated a hydrophobic surface. It is clear that the contact angle of the Control decreased rapidly during the first three seconds compared with the treated samples, and eventually approached a relatively stable value around 2–3°. In addition, the contact angles of treated wood were much larger, even after 60 s without a significant reduction, which suggests that alkali lignin improves the surface hydrophobicity of wood due to its hydrophobic property. Another reason for this effect could be that the alkali lignin existing on the wood surface may create wood roughness. The contact angle increased with increasing heating temperature at a given testing time. Specifically, the contact angle of AL 180 °C exceeded 140°, which almost corresponds to a super-hydrophobic surface with a water contact angle greater than 150° [27]. The surface active functional groups of the wood probably reduced after heat treatment [28], and high temperature may promote the reactions between surface functional groups and alkali lignin.

### 3.6. Moisture Adsorption and Water Absorption

**Figure 6:**
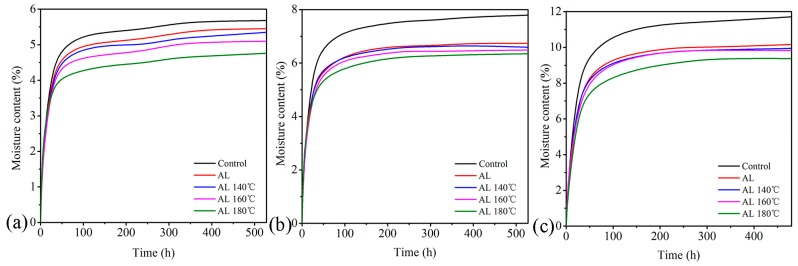
Moisture adsorption processes under (**a**) 45% relative humidity (RH), (**b**) 55% RH, and (**c**) 72% RH at 25 °C for the samples.

Figure 6 presents the adsorption processes at three RH conditions for the samples. Hygroscopicity of samples subjected to heat treatment at 180 °C for 2 h without lignin impregnation was also investigated as a reference, but the results indicated a very limited improvement and are not discussed here. Among the five groups in the Figure 6, the Control exhibited the highest MC, followed by AL, AL 140 °C, AL 160 °C, and AL 180 °C. Taking the 72% RH as an example, the equilibrium MC of AL, AL 140 °C, AL 160 °C, and AL 180 °C dropped by 13.2%, 15.2%, 15.4%, and 20.0% compared with the Control, respectively, which suggests that alkali lignin impregnation combined with heat treatment can reduce wood hygroscopicity. This can be explained by the small molecular alkali lignin reacting with –OH in the wood cell wall (Figure 3) and heat treatment led to a lower moisture adsorption due to the reduced hygroscopicity resulting from hemicellulose degradation.

The 18/W calculated by the H–H sorption model is listed in Table 1. All the values of 18/W for the samples fell in the range of 4% to 6%. When the effective –OH of the sample reacted with the monolayer adsorbed water, the MC should be limited to 4% to 6%. This result is consistent with previous research [29]. A comparison of 18/W among different samples shows that the value of the modified samples decreases compared with the Control. It also verifies a reduction in available sorption sites after alkali lignin plus heat treatment.

**Figure 7:**
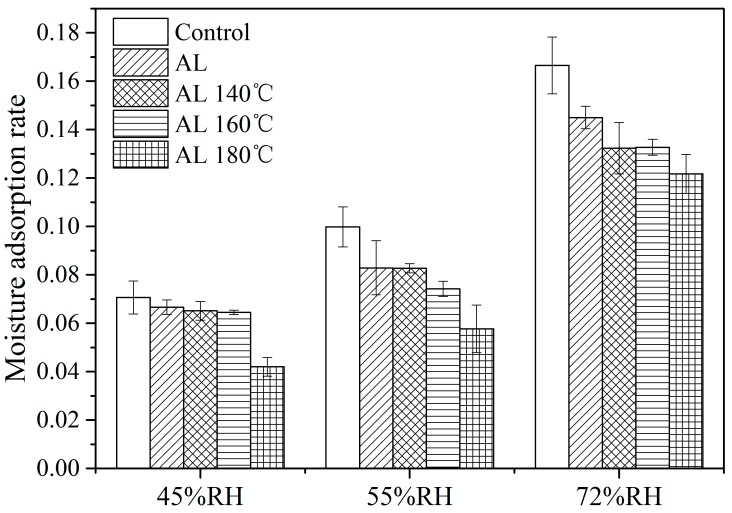
Moisture adsorption rate of the samples during the first 24 h.

The moisture adsorption rate of the samples was evaluated by taking the first derivative according to Figure 6. The corresponding results of the first 24 h during the adsorption processes are shown in Figure 7. From the figure, the adsorption rate of the samples increased with increasing RH. The adsorption rate of alkali lignin-heat treated samples declined compared with the Control. Specifically, the values of AL, AL 140 °C, AL 160 °C, and AL 180 °C dropped by 16.9%, 17.1%, 25.7%, and 42.2%, respectively, under the 55% RH condition compared with the Control. This implies that alkali lignin plus heat treatment decreases not only moisture adsorption amount but also adsorption rate for the samples, which may due to the blocking of water pathways caused by alkali lignin (Figure 1).

**Figure 8:**
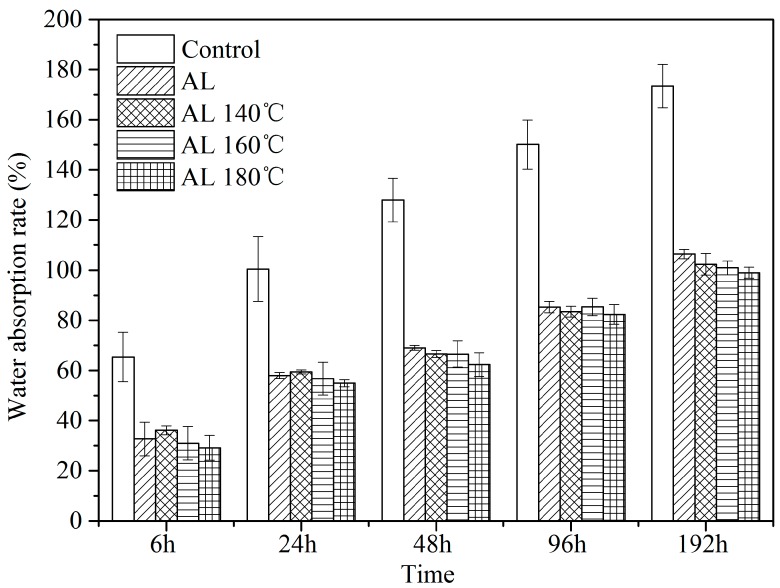
Water absorption rate of the samples as a function of immersion time.

As shown in Figure 8, the WAR increased with immersion time. The rate of alkali lignin-modified samples showed an apparent reduction compared with the Control. After 192 hours’ immersion, the WAR of the alkali lignin-modified sample was 65–75% lower than that of the Control. This result indicates that alkali lignin has a remarkable waterproofing effect, owing to the filling effect in the cell cavity of wood fibers or vessels and the blockage effect in the pit caused by alkali lignin (Figure 1). Therefore, water transportation was inhibited. High temperature treatment further decreased WAR slightly, and the degradation of hydrophilic hemicelluloses during thermal treatment could be responsible for this phenomenon, as stated above.

### 3.7. Dimensional Stability

**Figure 9:**
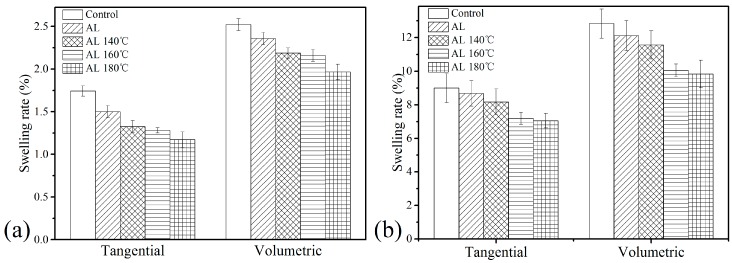
Tangential and volumetric swelling rate of the samples: (**a**) through moisture adsorption (54% RH) and (**b**) through water absorption after 20 days’ immersion.

Figure 9 presents the difference in swelling rate between moisture adsorption and water absorption. In Figure 9a, both the tangential and volumetric swelling rates of AL-treated wood decreased compared with the Control, and the swelling rate reduced more apparently as heat temperature increased. Concerning the tangential swelling rate through moisture adsorption, for example, the values for AL, AL 140 °C, AL 160 °C, and AL 180 °C dropped by 13.8%, 23.8%, 26.4%, and 32.6% compared with the Control, respectively. For swelling rate through water absorption (Figure 9b), the alkali lignin penetration with heat-treatment clearly lowered the tangential and volumetric swelling rates. These results were predictable as both hygroscopicity and WAR decrease as mentioned above (Figure 6 and Figure 8). The swelling of wood could be restrained by alkali lignin plus heat treatment so that the dimensional stability improved. When hydrophobic material like alkali lignin bulks the cell, the water affinity of wood reduces, and the space available for the water molecules to enter the cell wall decreases, which leads to reduced swelling. The results can also be related to the fact that heat treatment at high temperature reduces wood hygroscopicity so as to the long-term dimensional stability improved [30].

### 3.8. Compressive Strength

Compression strength values of the samples listed in Table 1 illustrate that the σ value for AL increased slightly compared with the Control and a more obvious improvement was observed as heat treatment temperature increased. This could be interpreted as an enhancement in the mechanical performance achieved by the combined modification. Alkali lignin acts as a stiffener while filling the pore spaces of wood [31], accounting for the increase in compressive strength after impregnation. A negative effect on the mechanical properties of the samples was not found for heat treatment samples. Conversely, the redistribution of alkali lignin and new chemical bonds formed between the alkali lignin and wood matrix benefited from high temperature treatment would further support the improvement in mechanical properties.

## 4. Conclusions

In this study, we reported an effective, low cost, and sustainable modification method for fast growing wood. The samples modified by alkali lignin combined with heat treatment produced obvious improvement both in hydrophobic properties and dimensional stability, without adversely changing the mechanical properties of the wood. Based on a multiscale modification of the cell cavity as well as cell wall levels, a novel wood with enhanced performance can be used as a fully green product, which can be easily disposed of and used as an ecologically friendly material. Since most chemical modifications are often hindered by ecological drawbacks, such as lack of sustainability, ecoefficiency, and recyclability of the products, this process promotes the efficient and sustainable development of the forestry industry.

## Figures and Tables

**Table 1:** Weight percent gain (WPG), mass loss, leaching rate, initial contact angle, 18/W, and compression strength (σ) of the samples. Number in parenthesis represents standard deviation from several replicates.

Sample	WPG (%)	Mass Loss (%)	Leaching Rate (%)	18/W (%)	σ (MPa)
Control	-	-	-	5.4 (0.1)	41.9 (1.3)
AL	12.3 (0.3)	-	3.3 (0.7)	5.3 (0.1)	42.5 (1.2)
AL 140 °C	12.4 (1.0)	0.8 (0.1)	2.3 (0.2)	5.2 (0.1)	43.8 (1.4)
AL 160 °C	11.2 (0.9)	1.2 (0.1)	1.9 (0.3)	5.1 (0.1)	45.4 (1.2)
AL 180 °C	11.8 (0.7)	2.2 (0.2)	1.4 (0.1)	4.9 (0.0)	45.6 (1.1)

**Table 2:** Lignin index (LI) for the Control, AL, and AL 180 °C samples.

Sample	LI_1263_	LI_1427_	LI_1507_	LI_1632_
Control	49.0%	52.8%	39.3%	73.2%
AL	78.6%	73.2%	72.1%	86.0%
AL 180 °C	55.2%	60.6%	48.8%	139.4%
